# Development of an IMU-Based Post-Stroke Gait Data Acquisition and Analysis System for the Gait Assessment and Intervention Tool

**DOI:** 10.3390/s25071994

**Published:** 2025-03-22

**Authors:** Yu-Chi Wu, Yu-Jung Huang, Chin-Chuan Han, Yuan-Yang Cheng, Chao-Shu Chang

**Affiliations:** 1 Department of Electrical Engineering, National United University, Miaoli 36003, Taiwan; yujungzip@gmail.com; 2 Department of Computer Science and Information Engineering, National United University, Miaoli 36003, Taiwan; cchan@nuu.edu.tw; 3 Department of Physical Medicine and Rehabilitation, Taichung Veterans General Hospital, Taichung City 40705, Taiwan; rifampin@gmail.com; 4 Department of Information Management, National United University, Miaoli 36003, Taiwan; cschang@nuu.edu.tw

**Keywords:** post-stroke gait assessment, inertial measurement unit, machine learning, gait assessment and intervention tool

## Abstract

Stroke is the fifth leading cause of death in Taiwan. In the process of stroke treatment, rehabilitation for gait recovery is one of the most critical aspects of treatment. The Gait Assessment and Intervention Tool (G.A.I.T.) is currently used in clinical practice to assess the gait recovery level; however, G.A.I.T. heavily depends on physician training and clinical judgment. With the advancement of technology, today’s small, lightweight inertial measurement unit (IMU) wearable sensors are rapidly revolutionizing gait assessment and may be incorporated into routine clinical practice. In this paper, we developed a gait data acquisition and analysis system based on IMU wearable devices, proposed a simple yet accurate calibration process to reduce the IMU drifting errors, designed a machine learning algorithm to obtain real-time coordinates from IMU data, computed gait parameters, and derived a formula for G.A.I.T. scores with significant correlation with the physician’s observational scores.

## 1. Introduction

Stroke is a major global health issue, affecting 15 million people annually and causing significant mortality and disability [1,2]. In Taiwan, it is the fifth leading cause of death, a primary cause of adult disability, and a significant burden on healthcare resources. Rehabilitation is key to stroke treatment. Based on the literature [3], the initial three months following a stroke are often referred to as the golden period for rehabilitation. During this crucial time, actively participating in a variety of therapeutic exercises can significantly reduce the physical impairments caused by the stroke. These impairments can affect stroke patients to varying degrees, depending on the severity and location of the brain injury, such as minor difficulties with coordination or balance for mild impairment, the difficulty of walking without assistance or using mobility aids for moderate impairment, and complete inability to walk without extensive support or assistive devices for severe impairment. Rehabilitation efforts during this golden period may enable some individuals to regain normalcy and lead independent lives. During the rehabilitation, clinicians use standardized stroke scales to quantify damage, including neurological deficits and gait abnormalities. Post-stroke patients often exhibit altered gait patterns with deviations in spatiotemporal characteristics [4], including reduced walking speed, shorter strides, increased energy expenditure, and asymmetry with uneven step lengths, stance times, and swing times between the affected and unaffected sides, impairing mobility and quality of life. While many regain walking ability, their gait remains biomechanically different from healthy individuals. For example, the right foot’s step length and stride length may differ from the step length and stride length of the left foot for a post-stroke patient. However, the step length and stride length of both feet may be similar for a healthy individual. Analysis of the time–distance parameters in patients who have had strokes also reveals that they walk more slowly than healthy age-matched subjects, and the amplitude of peak values of lower extremity joints is reduced for patients who have had strokes [5]. Proper treatment and therapy can help optimize mobility.

Identifying clinical features related to post-stroke walking ability is crucial for developing effective gait training programs. Gait recovery has consistently been recognized as a primary rehabilitation goal, especially since impairments in muscle strength, motor function, and balance have been observed to be highly correlated with walking ability. The strength of affected hip flexors, ankle plantar flexors, knee extensors, and unaffected knee flexors and ankle plantar flexors is moderately to highly correlated with walking and stair-climbing speed (r = 0.5~0.8) [6,7] (moderate correlation: r = 0.5~0.7, indicating a noticeable relationship between two variables; high correlation: r = 0.7 to 0.9, suggesting a much stronger association where the variables are closely aligned). The Fugl-Meyer assessment of affected lower limb motor function significantly correlates with gait speed in mild to moderate stroke patients (r~0.6) [8]. Balance function measured by the Berg Balance Scale is highly correlated with functional walking distance [6-minute walk test (6 MWT) and 12-minute walk test (12 MWT)] at self-selected speeds (r = 0.78–0.80) [9].

In clinical gait assessment, a person’s walking “ability” and “how” they walk are relevant. A stroke patient’s walking ability is a function of stroke severity, typically based on two main aspects: how far a person can walk and their endurance. These are usually assessed using 3-minute, 6-minute, or 10-minute walk tests [1]. Functional Ambulation Category (FAC), Short Physical Performance Battery (SPPB), and/or Motor Assessment Scale (MAS) can also be used for further evaluation. On the other hand, assessing gait quality or “how” one walks involves studying gait patterns and specific gait characteristics. Historically, gait characteristics were more often studied in research settings rather than clinical environments. Still, instrumented gait analysis, including kinematic and kinetic assessments, can be used today to help clinicians establish patient-specific quantitative functional mobility benchmarks and goals.

In the early stages of post-stroke recovery, doctors perform qualitative observations, also known as visual gait analysis (using the naked eye or video), to assess gait performance and functional improvement [10]. Current clinical assessment methods based on visual observation largely rely on training and clinical judgment. Despite being scrutinized for inter-observer variability [11], “observational gait analysis” methods remain popular among clinicians due to their simplicity, availability, and low cost [12]. However, these qualitative analyses’ validity, reliability, specificity, and responsiveness [13,14] have been questioned [11]. Overall, subjective observational gait scales may be useful for preliminary assessment of some spatiotemporal and/or kinematic gait parameters but are insufficient for analyzing the multifaceted nature of gait variability and complexity (e.g., motor and balance parameters). The timing for more complex gait analysis in stroke patients may depend on stroke severity and other factors such as fatigue, instability, pain, and persistent poor walking patterns despite adequate muscle activity during passive examination [1]. Nishi et al. [15] highlighted the importance of evaluating gait speed adjustability and instability in free-living environments to understand how individuals with post-stroke dysfunction navigate diverse contexts. They studied 50 stroke survivors wearing accelerometers on their lumbar spine (L5) for 24 h in real-life conditions. Directed acyclic graphs (DAGs) were generated using gait parameters derived from the acceleration data, and clustering analyses identified three distinct patterns based on four key variables: Ashman’s D (step velocity), Fugl-Meyer Assessment, step time asymmetry, and step length. The findings underscore the diversity in gait control strategies among stroke survivors, suggesting the need for tailored rehabilitation approaches to address individual variations in gait patterns. Faisal and Deen [16] introduced a simplified gait index based on critical parameters—walking speed, maximum knee flexion angle, stride length, and stance-swing phase ratio—to assess overall gait quality. They used a gait dataset of 120 healthy individuals from two gait datasets [17,18] and found the healthy gait index range (0.5–0.67). A support vector machine algorithm was applied to validate this index, achieving approximately 95% classification accuracy. The study also confirmed the index’s reliability and effectiveness by comparing it with other published datasets, which aligned with the predictions.

Since the 1990s, instrumented gait analysis has become the standard in research, providing an accurate and reliable biomechanical gait assessment method, including key parameter data (spatiotemporal data measurements and kinematic and kinetic measurements) [5,19,20]. However, gait laboratories typically include large, cumbersome, and expensive equipment such as motion capture systems (Vicon, Motion Analysis Inc., Qualisys, OptiTrack, etc.), force plates (Bertec, Kistler, Noraxon, etc.), instrumented walkways (Tekscan, GaitRite, etc.), balance platforms, and other instruments considered important for specific research applications (including post-stroke gait assessment) [1]. Among these devices, motion analysis systems (kinematic systems) are used to obtain the magnitude and timing of individual joint motion. For this purpose, the position and orientation of the limb segments and the join angles are recorded, using electrogoniometers or video cameras. The force platform or plate (kinetic system) provides a full three-dimensional description of the average ground reaction force vector beneath the foot, including three components of force (vertical, lateral (side-to-side), and fore-aft), the two coordinates of the center of pressure, and the moments about the x, y, and z axes. The construction and configuration of these laboratories are specific, aiming to conduct accurate tests, for example an area at least 9 m wide, 11 m long, with 3–5 m high ceilings (higher for stair climbing and motion applications) and special flooring. Therefore, it is not surprising that these setups, possibly only found in laboratory environments, are uncommon in clinical settings due to construction challenges and costs, as well as the lack of appropriately skilled clinicians to operate and manage/interpret the large amounts of data generated from various sensing technologies used in post-stroke gait rehabilitation.

With technological advancements, today’s small, lightweight wearable IMU sensors are rapidly revolutionizing gait assessment in research environments and have the potential to be incorporated into routine clinical practice. These sensors can generally be fixed to specific body parts using straps and, with enhanced wireless communication capabilities, can detect real-time synchronized information from multiple devices worn on limb joints during walking activities, thereby monitoring movement changes and assessing gait performance [21]. They provide researchers with new opportunities for continuous gait recording, offering dynamic methods for quantifying gait over time and providing real-time feedback to patients and clinicians. Their lightweight, portability, and low-cost offer research potential for data collection outside the laboratory and in natural environments (clinics, sports fields, etc.). Moreover, these sensors can easily synchronize with other physiological measurement devices (such as EMG, ECG, and EEG), providing valuable multifactorial continuous data on subjects/patients in various environments, better reflecting the true living experiences and functional walking conditions in community settings [22].

IMUs, through IC (integrated circuits) processing and MEMS (micro-electromechanical systems) technology, are typically just a few millimeters in size, like 2–5 mm per side, and have functions such as accelerometers, gyroscopes, and sometimes magnetometers, capable of measuring and reporting information about an object’s speed, acceleration, vector, and gravity. In principle, IMUs derive a series of data calculations based on Newton’s mechanics formulas. Therefore, given known physical mass and force, applying the F = M × a rule allows for calculating acceleration values. Using advanced mathematical concepts, integrating acceleration yields speed, and similarly, integrating speed yields displacement results in corresponding three-axis coordinates. Thus, once the basic signals are detected by the accelerometer and appropriate algorithms are selected for filtering and analysis, we can practically capture relevant gait variables during the walking period, even covering estimated values such as step frequency and stride length [21].

With the advent of small, lightweight wearable measurement devices, significant progress has been made in processing gait data. Artificial intelligence (AI), including machine learning (ML), provides data analysis and model building, effectively performing recognition and prediction [23,24,25,26,27]. Reference [26] used dynamic gait data, employing SVM (support vector machine), RF (random forest), and ANN (artificial neural network) to classify young adults, older adults, and elderly patients, using KPCA (kernel principal component analysis) for data dimensionality reduction in the SVM classifier. Reference [24] used SVM, ANN, and RBFNN (radial basis function neural networks) classifiers to distinguish different walking conditions in hemiplegic patients, finding that a SVM had a higher recognition accuracy. Reference [23] used a general regression neural network (GRNN) as a decision tool, and reference [25] used an optimized ANN to identify hemiplegic stroke and able-bodied ambulation. Reference [28] described the characteristics of hemiplegic gait using a PCA (principal component analysis) of trunk movement (TM) and gait event (GE) parameters from IMUs. Reference [29] analyzed different IMU-based gait parameters proposed in the literature to assess frailty status (robust, pre-frail, or frail) or fall risk, showing that the most important parameters for frailty diagnosis were double support time, gait speed, stride time, step count, and steps per day or walking percentage per day. In the case of fall risk detection, the most relevant parameters were those related to trunk stability or motion. Luo et al. [30] proposed a gait analysis algorithm designed for heel-mounted IMUs to accurately assess heel dynamics and compute a comprehensive range of spatiotemporal gait parameters and parameters related to symmetry and variability. Three IMUs were mounted on a shoe: one on the instep, one in the center of the insole with a cavity, and one on the heel. Experiments included straight walking and daily activity simulations, with an optical motion capture (OMC) system as a reference. Results showed strong correlations (r > 0.9) and high reliability for all parameters (minimum intraclass correlation coefficient of 0.921). Lin et al. [31] addressed the growing issue of unbalanced walking among older adults by developing a deep learning (DL) model to improve balance assessment methods. The proposed model combines a convolutional neural network (CNN) with a long short-term memory (LSTM) network to predict scores for three balance scales: Berg Balance Scale (BBS), Timed Up and Go (TUG), and Single-Leg Stance (SLS). Gait data from 15-meter walks were collected using seven IMUs (on the lumbar spine, right upper arm, left upper arm, right thigh, left thigh, right calf, and left calf), with the CNN-LSTM model achieving accurate predictions for BBS and TUG scores. BBS and TUG assessments require only two IMUs placed on the thighs, producing mean absolute errors (MAEs) of 1.2562 and 1.4016 s, respectively. While the SLS showed an average MAE of 20.8886. Testing also demonstrated that using just one IMU on the right calf for BBS and TUG yielded comparable results, with MAEs of 1.4334 and 1.5229 s. Rokhmanova et al. [32] explored the impact of kinematic errors on learning therapeutic gait using vibrotactile feedback for knee osteoarthritis patients. The accuracy of inertial measurement units (IMUs) was compared to marker-based motion capture data, revealing that higher tracking errors resulted in slower learning rates and more incorrect responses to feedback. Participants with lower tracking errors showed better retention of learned gait in an outdoor setting compared to the control group, while those with higher errors did not. Tracking errors were correlated with foot size and angle magnitude, indicating potential bias. Guo et al. [33] introduced a temporal convolutional network (TCN)-based approach for robust and automatic gait phase recognition across various scenarios and walking speeds. Data from real-world overground walking experiments and public treadmill datasets were used to validate the method. In the overground walking experiment, two IMU sensors mounted on the shank with velcro belts were used to obtain gait data. A force plate synchronized with a motion capture system (Mocap) was used as the gold standard in the gait analysis. The TCN approach achieved 97% accuracy in gait-phase estimation, outperforming six machine learning models (K-nearest neighbor, naïve Bayes, support vector machine, decision tree, logistic regression, and multilayer perception) and two neural network models (fully convolutional network and long short-term memory).

In summary, the effectiveness of gait recovery in post-stroke rehabilitation is crucial. Collecting gait parameters during rehabilitation and daily life can help medical teams establish patient-specific quantitative functional mobility benchmarks and goals. Lightweight wearable devices can assist patients in daily rehabilitation and walking training at home, which is meaningful for clinical treatment.

In this paper, we used wearable devices to collect six-axis data on stroke patients’ walking gait (XYZ three-axis linear accelerations and XYZ three-axis angular velocities). We also designed a calibration process and a machine learning model to obtain the required gait parameters and proposed a formula for assessing the G.A.I.T. score. These scores and the doctor’s observational scores were evaluated through statistical analysis to find their correlations. The contributions of this work are summarized as follows.

- Development of an IMU-Based Gait Data Acquisition System: Proposed a wearable device based on inertial measurement units (IMUs) that can collect real-time gait data from stroke patients, overcoming the limitations of traditional gait assessment tools;
- Simple and Accurate Calibration Process: Designed a straightforward calibration method to mitigate drift errors in IMU data, ensuring the accuracy and reliability of the measurements;
- Application of Convolutional Neural Networks (CNNs): Utilized a CNN model for gait data analysis, enabling accurate prediction of gait parameters and providing real-time feedback;
- Analysis of Eight Stages of the Gait Cycle: Clearly defined the eight stages of the gait cycle and developed a scoring formula based on these stages, enhancing the objectivity of gait assessment;
- High Correlations with Physician Observations: Demonstrated significant correlations between G.A.I.T. scores calculated from IMU data and those assessed by physicians, validating the system’s effectiveness;
- Clinical Application Potential: Provided a tool for gait training in daily life, assisting healthcare teams in establishing patient-specific functional mobility benchmarks, thereby improving recovery outcomes for stroke patients;
- Advancement of Gait Assessment Technology: Promoted the application of gait assessment technology in clinical practice through small, lightweight wearable devices, offering new directions for future research and practice.

## 2. Materials and Methods

**Figure 1:**
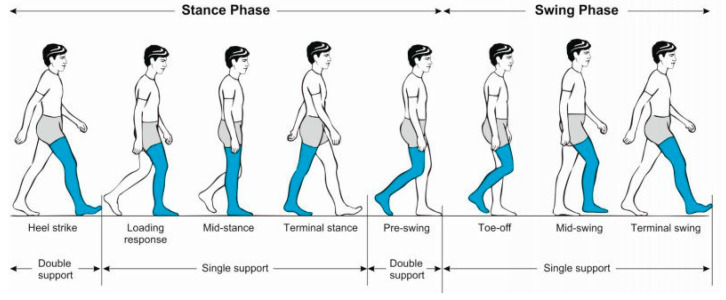
Gait cycle (GC) [34].

In gait data collection, it is necessary to identify the various phases and parameters of the human gait cycle (GC), as shown in Figure 1 [34]. Eight states are required to complete a gait cycle: heel strike (HS) (initial contact), loading response, mid-stance, terminal stance, pre-swing, toe-off (initial swing), mid-swing, and terminal swing.

### 2.1. IMU Sensor

**Figure 2:**
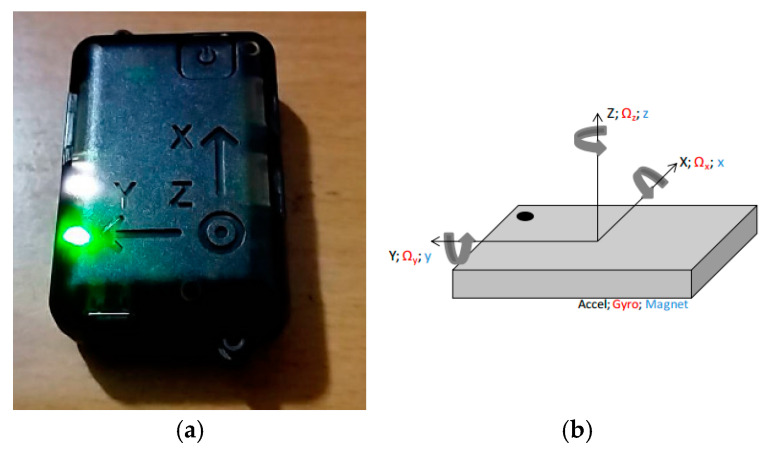
(**a**) NGIMU; (**b**) schematic diagram of the nine-axis sensor coordinate system.

This paper used NGIMU [35] as the data acquisition device to facilitate gait data collection and ensure data stability. The dimensions of the NGIMU are 56 × 39 × 18 mm, and it weighs 46 g. The appearance is shown in the left image of Figure 2. The right image of Figure 2 is a schematic diagram of the nine-axis sensor coordinate system, where the *x*-axis points forward, the *y*-axis points to the left, and the *z*-axis points upward (towards the sky). The definition of rotational angular velocity is based on the right-hand rule, where the thumb points along the axis, and the direction of the curled fingers defines the positive rotational velocity.

NGIMU has a built-in Attitude and Heading Reference System (AHRS) algorithm, which calculates and provides data types and related static accuracy values. The data types include quaternion, rotation matrix, Euler angles, linear acceleration, and earth acceleration. NGIMU measurement data can be transmitted via a USB wired connection or WiFi/UDP wireless connection.

**Figure 3:**
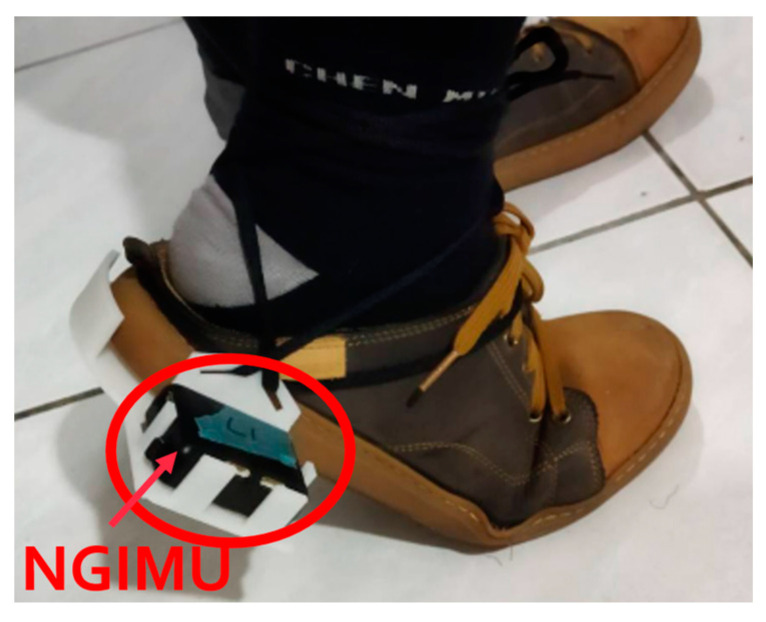
NGIMU in the fixture at the heel of the shoe.

**Figure 4:**
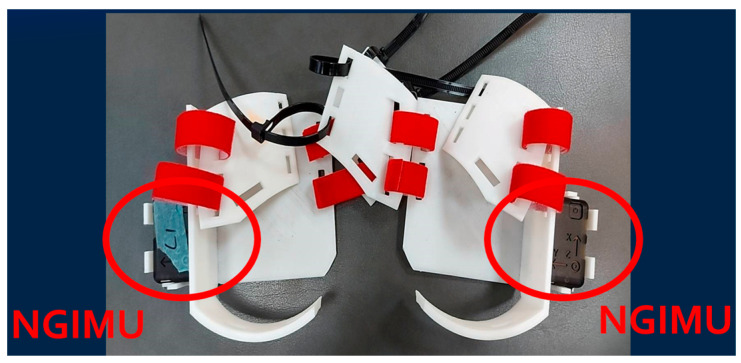
3D-printed fixtures for placing the NGIMU.

To accurately measure the height of the shoe sole from the ground during actual walking, we do not attach the NGIMU to the ankle. Instead, we designed a wearable fixture for the heel of the shoe, placing the NGIMU sensor in a fixed position, as shown in Figure 3. This avoids the height error caused by attaching it to the ankle and provides a position and angle closer to the sole. Figure 4 shows the 3D-printed fixture for placing the NGIMU (for both left and right feet).

### 2.2. Data Analysis Procedure

**Figure 5:**
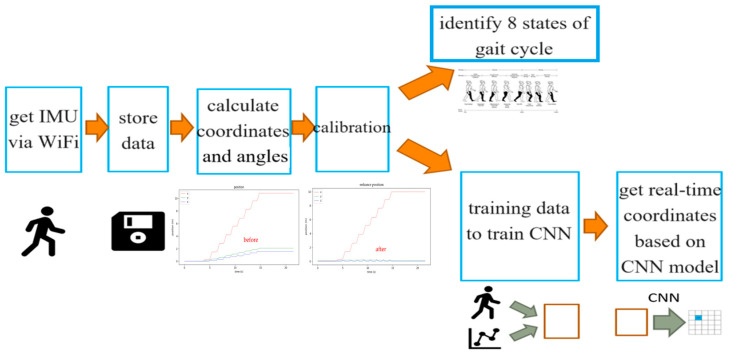
Data analysis process.

Figure 5 illustrates the data analysis process. Using WiFi, the data measured by the NGIMU (si*x*-axis data and Euler angles) is saved to a computer in Excel format. These data are then converted into XYZ spatial coordinate distance values, with the *x*-axis representing forward distance, the *Y*-axis representing left (+) and right (−) deviation during forward movement, and the *Z*-axis representing heel height. The calibration procedure is used to calibrate the XYZ distance measurements. The calibrated values can determine the occurrence points of the GC’s eight states and serve as labels for supervised learning, training a model to map the original six-axis data to XYZ spatial distance values. This paper uses a convolutional neural network (CNN) model to train the data.

### 2.3. Three-Axis XYZ Distance Calibration

**Figure 6:**
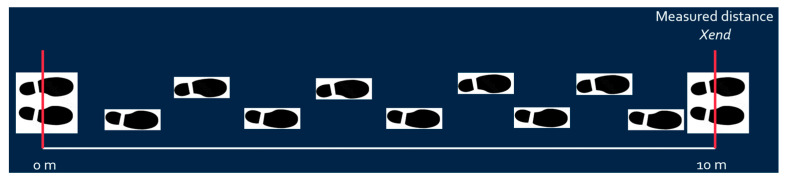
*X*-distance calibration.

**Figure 7:**
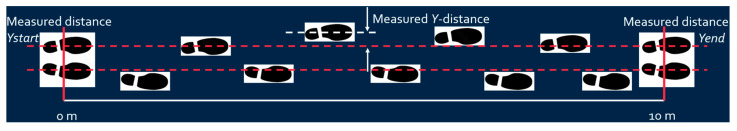
*Y*-distance calibration.

**Figure 8:**
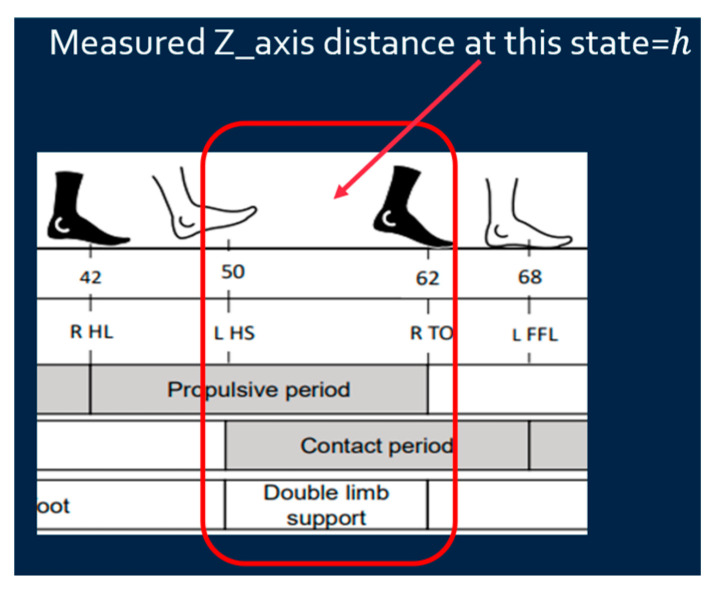
*Z*-distance calibration.

Although the NGIMU has good static angular velocity accuracy, it was found that the calculated spatial XYZ three-axis distance values have cumulative integration errors, resulting in data drift. Therefore, calibration is necessary. Typically, precise calibration requires expensive optical motion capture (OMoCap) systems, which need multiple optical cameras and a setup space. However, due to budget constraints and practical site space, a simple yet relatively accurate calibration procedure is designed to be suitable for gait data. The procedure is as follows:

1. First, fix the walking *x*-axis linear distance (e.g., ten meters for post-stroke patient to walk), mark the starting and ending lines, and ideally draw a straight line between the starting and ending lines. The subject should walk between the starting and ending points;
2. *X*-axis distance calibration: The predetermined *x*-axis length value (e.g., 10 m) calibrates the calculated x distance based on Equation (1) as shown in Figure 6.
  (1) $$X_{cali}=\frac{10}{Xend}\times X_{meas}$$
  where $X_{meas}$ is the measured *x*-axis distance obtained from IMU, Xend is the measured *x*-axis distance at the end point, and $X_{cali}$ is the calibrated *x*-axis distance;
3. *Y*-axis distance calibration: Set the Y distance to zero when both feet stand at the starting point. Assume the Y distance at the end of the walk is approximately the same as at the starting point (since walking along the *x*-axis line), and calibrate the Y-distance accordingly based on Equation (2) as shown in Figure 7.
  (2) $$Y_{cali}=Y_{meas}-(Yend-Ystart)$$
  where $Y_{meas}$ is the measured *Y*-axis distance obtained from IMU, Yend and Ystart are the measured *Y*-axis distances at the end and starting points, respectively, and $Y_{cali}$ is the calibrated *Y*-axis distance. Since the walking lane has starting and end lines, it is reasonable to assume the patient begins and finishes walking with both feet standing at the starting and end line. The measured Yend and Ystart are supposed to be the same, and the difference between these two values can be used to calibrate the measured *Y*-axis distance;
4. *Z*-axis distance calibration: Since walking is on a flat floor, the Z-distance when the foot is fully on the ground (stationary) is regarded as zero to calibrate the z-distance based on Equation (3) as shown in Figure 8.
  (3) $$Z_{cali}=Z_{meas}-h$$
  where $Z_{meas}$ is the measured *Z*-axis distance obtained from IMU, h is the measured *Z*-axis distance at double limb support state, and $Z_{cali}$ is the calibrated *Z*-axis distance. Since the *Z*-axis distance (foot height) is supposed to be zero at the double limb support state, the difference between the measured *Z*-axis distance at this state h and zero can be used to calibrate the measured *Z*-axis distance.

### 2.4. Determining the Eight States of the Gait Cycle

To identify the gait cycle, data from both feet are required. The analysis of the corresponding movement relationships is as follows (with the right foot as the primary test foot and the left foot as the secondary test foot):

1. Initial Contact (Heel Strike): The movement begins with the heel of the primary test foot touching the ground (start of first double support);
2. Loading Response: The end of the first double support with the heel of the secondary test foot at its highest point under the condition that the X-distance of the secondary test foot is almost not changing;
3. Mid-stance: The secondary test foot reaches the body’s center point (the secondary test foot position surpasses the primary test foot);
4. Terminal Stance: The heel of the secondary test foot touches the ground (start of the second double support);
5. Pre-swing: The end of the second double support phase with the heel of the primary test foot at its highest point under the condition that the X distance of the primary test foot is almost not changing;
6. Initial Swing (Toe-off): The highest point of the primary test foot (maximum knee flexion);
7. Mid-swing: The primary test foot is parallel to the floor;
8. Terminal Swing: The heel of the primary test foot touches the ground.

Based on the collected six-axis posture data, the criteria for determining each gait phase are as follows:

1. Initial Contact: The maximum point of the right pitch angle;
2. Loading Response: The minimum point of the left pitch angle;
3. Mid-stance: The left *x*-axis position surpasses the right *x*-axis position;
4. Terminal Stance: The maximum point of the left pitch angle;
5. Pre-swing: The minimum point of the right pitch angle;
6. Initial Swing: The maximum point of the right *Z*-axis height;
7. Mid-swing: The right pitch angle returns to zero;
8. Terminal Swing: The maximum point of the right pitch angle.

### 2.5. Convolutional Neural Network (CNN)

**Figure 9:**
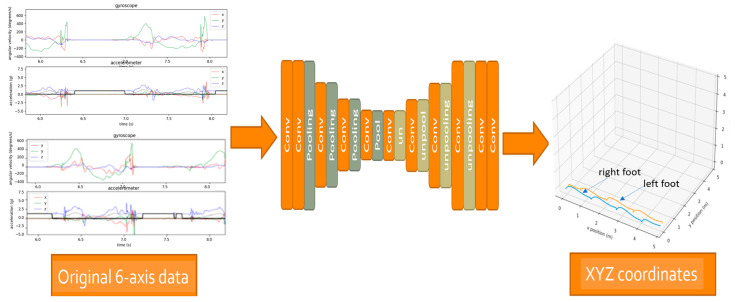
CNN for XYZ coordinates.

Convolutional Neural Networks (CNNs) are primarily used for processing and analyzing data with a grid-like structure, such as images and videos. The uniqueness of CNNs lies in their use of convolutional layers, pooling layers, and flattening layers to automatically learn features from the input data. Figure 9 shows the schematic diagram of the CNN model used in this work. The model consists of 11 layers with a kernel size of 41. The data are processed with convolutions and pooling before being upscaled to predict coordinate positions. Each training data set has a length of 1392 points, with each point containing six-axis data (*XYZ*-axis linear acceleration values and *XYZ*-axis angular velocity). The output data are 3D coordinate positions (XYZ three-dimensional position coordinates).

### 2.6. Gait Assessment and Intervention Tool (G.A.I.T.)

To accurately assess the gait of a post-stroke patient, we need gait coordination measurement and force production, including gait kinematics and gait kinetics, customarily measured using motion capture and force plate systems in well-equipped gait research laboratories. However, in clinical practice and certain gait research settings, the access to such technology is often unavailable. Additionally, the time constraints of clinical practice make it impractical to use this instrumented technology in its current form due to the labor-intensive process of data collection and analysis. However, observational gait coordination measures can be employed in these cases [36]. The Gait Assessment and Intervention Tool (G.A.I.T.) is a 31-item measure designed to evaluate the coordinated movement components of gait and identify associated gait deficits. It offers several advantages, including a comprehensive, objective-based scoring method and the ability to incrementally measure improvement within specific items. An inexperienced clinician, after receiving training, achieved an inter-rater reliability with an experienced rater of an intraclass correlation coefficient (ICC) equal to 0.99 [10]. The G.A.I.T. effectively distinguished between two gait training interventions, demonstrating an additional benefit of functional electrical stimulation (FES) compared to similar comprehensive gait training.

**Figure 10:**
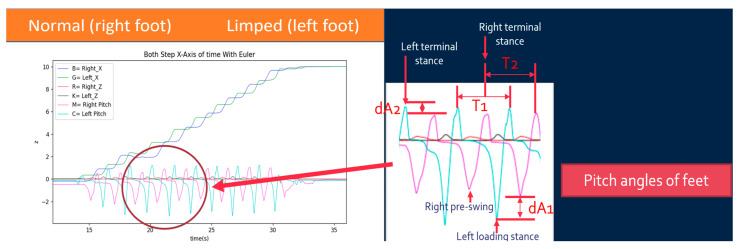
Parameters of GC’s states for G.A.I.T.

In this paper, we used simple IMU-based wearable devices to collect gait data of post-stroke patients and derive G.A.I.T. scores based on the GC’s state parameters. These scores are compared with those evaluated by the patients’ doctor. The parameters of GC’s states used for G.A.I.T. score are the pitch angles of the left terminal stance, right terminal stance, left loading stance, and right pre-swing and the times associated with these states, as shown in Figure 10 where the limped foot of the patient is left foot. The following variables are defined and used for calculating G.A.I.T. score based on Equations (4)–(20).

Maximum pitch angle of left foot step *i*: $P_{L,max,i},$
Minimum pitch angle of left foot step *i*: $P_{L,min,i},$
Maximum pitch angle of right foot step *i*: $P_{R,max,i},$
Minimum pitch angle of right foot step *i*: $P_{R,min,i},$

(4) $$\mathrm{Average}\;\mathrm{of}\;\mathrm{the}\;\mathrm{maximum}\;\mathrm{pitch}\;\mathrm{angle}\;\mathrm{of}\;\mathrm{left}\;\mathrm{foot}\;\mathrm{over}\;\mathrm{all}\;\mathrm{steps}:\;\bar{P_{L,max,i}}=e,$$

(5) $$\mathrm{Average}\;\mathrm{of}\;\mathrm{the}\;\mathrm{minimum}\;\mathrm{pitch}\;\mathrm{angle}\;\mathrm{of}\;\mathrm{left}\;\mathrm{foot}\;\mathrm{over}\;\mathrm{all}\;\mathrm{steps}:\;\bar{P_{L,min,i}}=f,$$

(6) $$\mathrm{Average}\;\mathrm{of}\;\mathrm{the}\;\mathrm{maximum}\;\mathrm{pitch}\;\mathrm{angle}\;\mathrm{of}\;\mathrm{right}\;\mathrm{foot}\;\mathrm{over}\;\mathrm{all}\;\mathrm{steps}:\;\bar{P_{R,max,i}}=g,$$

(7) $$\mathrm{Average}\;\mathrm{of}\;\mathrm{the}\;\mathrm{minimum}\;\mathrm{pitch}\;\mathrm{angle}\;\mathrm{of}\;\mathrm{right}\;\mathrm{foot}\;\mathrm{over}\;\mathrm{all}\;\mathrm{steps}:\;\bar{P_{R,min,i}}=h,$$

(8) $$\max\left(P_{L,max,i}\right)=c,$$

(9) $$\min(P_{L,min,i})=d,$$

(10) $$\max\left(P_{R,max,i}\right)=k,$$

(11) $$\min(P_{R,min,i})=m,$$

(12) $$\left|P_{L,max,i}-P_{R,max,i}\right|=dA_{2,i}$$

(13) $$\left|P_{L,min,i}-P_{R,min,i}\right|=dA_{1,i}$$

(14) $$\mathrm{Average}\;\mathrm{of}\;dA_{2,i}=a\;$$

(15) $$\mathrm{Average}\;\mathrm{of}\;dA_{1,i}=b$$

(16) $${Arange}_{L}=\left(e-f\right)$$

(17) $${Arange}_{R}=(g-h)$$

(18) $$p=\left|\bar{{T1}_{i}}-\bar{{T2}_{i}}\right|$$

(19) $$q=\frac{\left(\bar{{T1}_{i}}+\bar{{T2}_{i}}\right)}{2}$$

(20) $$G.A.I.T.Score=\frac{a+b+\left(c-e\right)+(k-g)+\left(f-d\right)+(h-m)}{{min(Arange}_{L}\;,{\;Arange}_{R})}\times (\frac{p}{q}+1)$$

The first term on the right-hand side in Equation (20) deals with spatial factors of the gait, while the second term on the right-hand side copes with temporal factors. The value of $dA_{2,i}$ is the difference between the maximum pitch angles of left and right feet for step *i*, and *a* is the average of $dA_{2,i}$. Therefore, the larger the value of *a*, the more asymmetric both feet are and, therefore, the higher the G.A.I.T. score should be. The same logic applies to variables *b* and $dA_{1,i}$. The value of *c* represents the maximum pitch angle of the left foot over all steps, and the value of *e* represents the average of the maximum pitch angles of the left foot over all steps. The difference between these two, (*c* − *e*), represents the walking consistency of the left foot. Therefore, the larger the value of (*c* − *e*), the less consistency the left foot is and the higher the G.A.I.T. score should be. The same logic applies to ( *k*− *g*). The minimum pitch angles can also be used to evaluate the walking consistency; therefore, (*f* − *d*) and (*h* − *m*) are used in Equation (20). The numerator of the first term on the right-hand side in Equation (20) may vary from person to person; the denominator of the first term on the right-hand side in Equation (20) is used to remove this factor. The smaller value from ${Arange}_{L}$ and ${Arange}_{R}$ is chosen as the denominator. $\bar{{T1}_{i}}$ and $\bar{{T2}_{i}}$ represent the average times of the strides of left foot and right foot, respectively. The value of *p* is the difference between $\bar{{T1}_{i}}$ and $\bar{{T2}_{i}}$. *p* is then divided by *q*, the average of $\bar{{T1}_{i}}$ and $\bar{{T2}_{i}}$. The smaller the value of *p*/*q*, the more consistent both feet are. The spatial term (the first term) on the right-hand side is adjusted using the second term (temporal factor *p*/*q* plus 1) on the right-hand side.

## 3. Results

The NGIMU measures data from both feet, sampling every 5 ms at a rate of 200 Hz and recording the position and angle of the feet. The stored data include time. The first batch of data was collected in a laboratory environment, with the walking distance along the *x*-axis set to 5 m. The dataset consists of 120 entries, with three participants (two males and one female, aged 19–21, with an average age of 20). After randomization, 80% of the data, 96 entries, was used as training data, and the remaining 24 entries were used as test data. Each data entry has a length of 1920 points (sampling frequency of 200 Hz, sampling time of about 10 s, including stationary time before and after walking).

The second batch of data were collected at Taichung Veterans General Hospital, Taiwan, with the walking distance along the *x*-axis set to 10 m. Currently, 26 data entries have been collected successfully. The collected data are stored in a local database and converted to CSV file format at the end of sampling. The files are divided into four parts, each containing six-axis acceleration data (excluding magnetic angle data due to their slow sampling rate) and Euler angle data for the left and right feet. The accelerometer data format is arranged as follows: the first column is time, the second to fourth columns are angular velocity, and the fifth to seventh columns are acceleration. The Euler angle data format is arranged with the first column being time, the second column being roll angle, the third column being pitch angle, and the fourth column being yaw angle.

### 3.1. XYZ Distance Calibration

**Figure 11:**
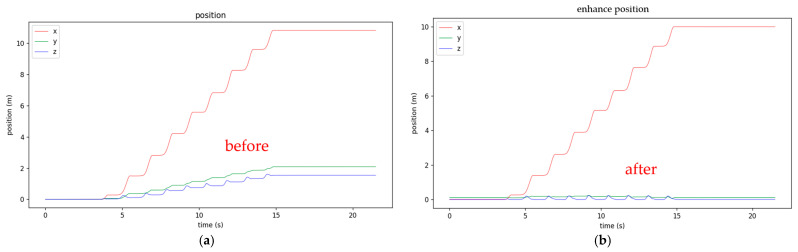
XYZ distance calibration: (**a**) before and (**b**) after calibration.

**Figure 12:**
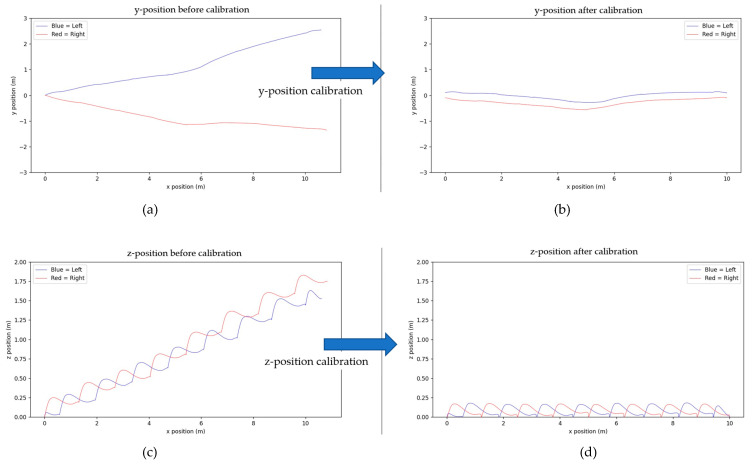
Close look of x- and z-positions before and after calibration. (**a**) y-position before calibration; (**b**) y-position after calibration; (**c**) z-position before calibration; (**d**) z-position after calibration.

After the data are collected and stored as a CSV file, they are calibrated using the proposed simple method. The *Y*-axis and *Z*-axis are adjusted based on the *x*-axis values, and the *x*-axis position is calibrated by setting the final distance to eliminate cumulative errors from integration. The trends of the XYZ distances before and after calibration are shown in Figure 11. The left figure shows that the calculated X distance from the built-in AHRS is not exactly 10 m due to integration errors. The shifts of Y-distance are also noticed. The Z-distance (height) gradually increases due to integration errors. The right figure shows the results after calibration, eliminating all the aforementioned phenomena. Figure 12 shows detailed comparisons for Z- and Y-distances before and after calibration. The upper figure of Figure 12 shows the calibration results of the Z-distance (height), where each stationary point is flat on the ground after calibration. The lower figure of Figure 12 shows the calibration results of the Y-distance, eliminating the difference in walking direction caused by the different foot angles before and during walking.

### 3.2. Eight States of Gait Cycle

**Figure 13:**
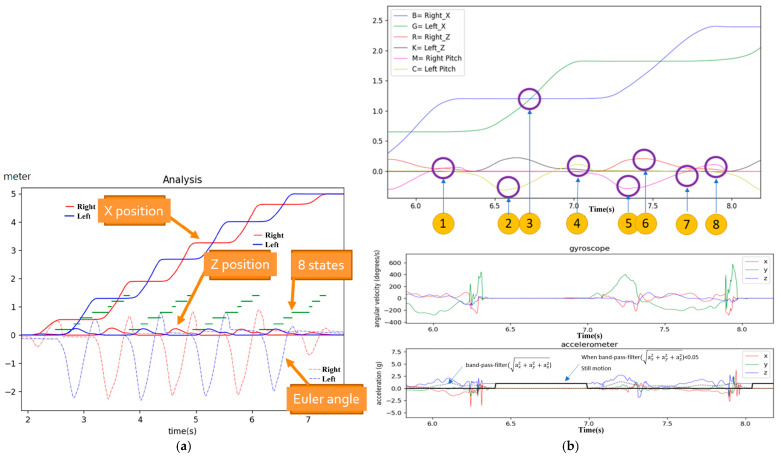
Determining the GC’s eight states. (**a**) Annotated time points of the eight states in each gait cycle; (**b**) top: x distance, z height, and pitch angle and bottom: gyroscope and accelerometer data.

Based on the criteria for determining the GC’s eight states, the corresponding results are shown in Figure 13. In the top graph of Figure 13b, blue represents the distance of the right foot along the *x*-axis (Right X), green represents the distance of the left foot along the *x*-axis (Left X), red represents the height of the right foot along the *Z*-axis (Right Z), black represents the height of the left foot along the *Z*-axis (Left Z), magenta represents the pitch angle of the right foot (right pitch), and cyan represents the pitch angle of the left foot (left pitch). The numbers within the solid circles indicate the time points of the eight states. The corresponding XYZ accelerations and angular velocities are shown in the bottom graph of Figure 13b. Figure 13a shows the successfully annotated time points of the eight states in each gait cycle. In this graph, the red solid/dash lines represent the primary test foot (right foot), and the blue solid/dash lines represent the secondary test foot (left foot). Therefore, red and blue solid lines illustrate the X-position and the *Z*-axis height, and the dashed red and blue lines represent the Euler angles (values are proportionally reduced). The green segments represent the eight states in the gait cycle (the eighth state is the starting point of the next cycle, so there are seven segments).

### 3.3. Convolutional Neural Network (CNN)

The 1D CNN is written in Python (version 3.8) and executed on the PyCharm (version 2024.1.6) platform using Pytorch (version 2.1.1+cu118). PySide2 (version 5.15.2.1) is chosen for GUI (Graphic User Interface). PyCharm is the development platform because it has better support for PySide2. PySide2 is similar to PyQt, making the development process equally simple. Additionally, PySide2 follows the LGPL license, allowing commercial use without being restricted by sharing regulations. Pytorch, an open-source AI development package since 2017, is known for its ease of use, quick model building, and excellent support for GPU computation, making it one of the most popular AI development tools in academia.

**Figure 14:**
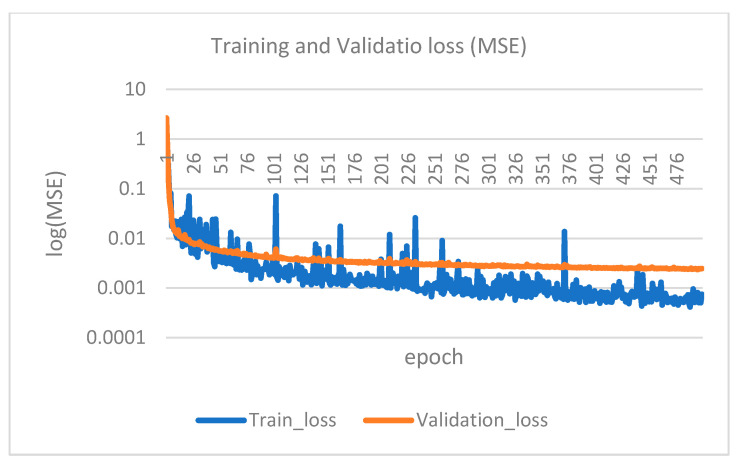
The training and validation loss (MSE).

**Figure 15:**
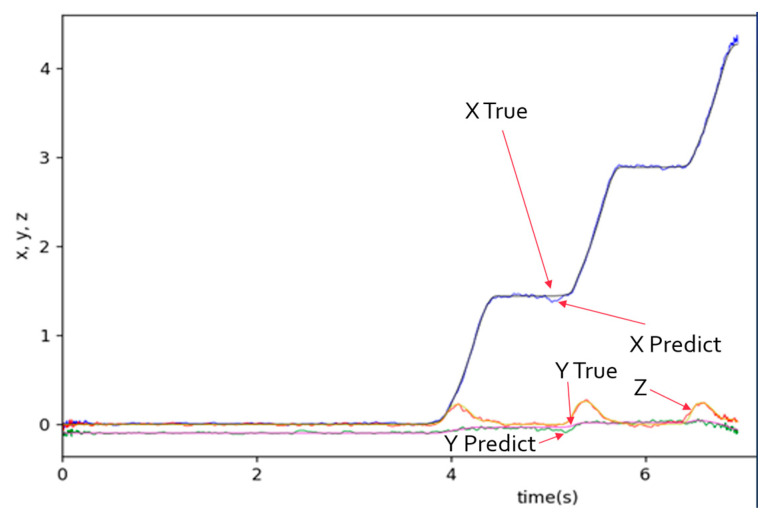
The prediction results after training.

The computer specifications for running the program are an Intel i7-12700H/2.30 GHz CPU (Santa Clara, CA, USA) and a Nvidia GeForce RTX 3060 GPU (Santa Clara, CA, USA). Training the 11-layer 1D CNN model with 96 data entries takes approximately 3 s per epoch. If only the CPU is used for training, it takes about 60–120 s. Figure 14 shows the training and validation loss (mean squared error, MSE) curve, and Figure 15 shows the prediction results after training with the epoch set at 500. The root of the MSE of the test data (24 data entries) is 5.42 cm, which is accurate enough for engineering purposes.

### 3.4. G.A.I.T. Score

Based on 26 data entries collected from the Taichung Veterans General Hospital (TVGH), Taiwan, Equation (20) calculates the G.A.I.T. scores for these data entries. Then, the calculated scores are compared with those evaluated by the director of the Rehabilitation Department of the TVGH. Table 1 lists these scores. Table 2 shows the mean and standard deviations of these two sets of scores. Table 3 shows the Pearson correlation of these two data sets with 0.752, which is significant at the 0.01 level (two-tailed). Table 4 shows the Spearman’s rho = 0.933 of these two data sets, which is also significant at the 0.01 level (two-tailed).

## 4. Discussion

The advantages of using NGIMU in this work include its excellent static angular velocity accuracy, which allows for more accurate distance measurements. It has built-in WiFi wireless transmission, making installation easy and requiring only a WiFi adapter without additional hardware. This feature makes collecting patients’ data convenient in the hospital environment. Additionally, NGIMU offers a user-friendly setup environment, making data collection more manageable for users.

NGIMU can measure nine-axis data using a gyroscope, accelerometer, and magnetometer; however, only six-axis data from NGIMU were used in this work. The sampling rates of the gyroscope and accelerometer both can reach 400 Hz, but the sampling rate of magnetic angles is only 20 Hz. We use 200 Hz as the sampling rate for measuring gait data, which is fast enough for walking movement.

Although NGIMU provides good static angular velocity and has a built-in AHRS algorithm, the accumulated error in calculating XYZ coordinates still exists, which can be observed in the left diagram of Figure 11. The diagram shows that the final X-distance exceeds 10 m, the final Y-distance is apart from the walking straight line by 2 m, and the Z-distances (height) of the GC’s double support are not zero (unrealistic floating above the floor). These errors make the gait data not practical to use. Therefore, a calibration process becomes essential. In this paper, a simple yet accurate enough calibration is proposed by using the special characteristics of 10 m linear walking. The calibration outcomes are satisfactory, which can be observed from the right diagram in Figure 11 and Figure 12. The final X-distance is at 10 m, the Y-distance is close to the walking straight line, and the Z-distance (height) is periodic and is zero at double support states.

**Figure 16:**
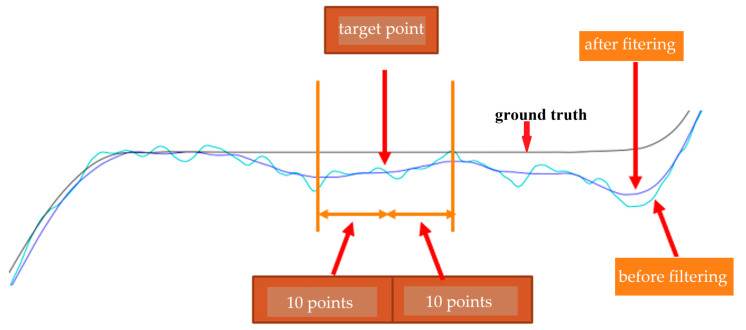
The filtered results.

By observing Figure 15, the predicted output results of the CNN model still exhibit some irregularities. We can further use a low-pass filter to filter the prediction results. In this paper, a filter based on the Slide Window method averages the data by extending 10 points to the left and right, effectively removing irregular waves. The filtered results are shown in Figure 16.

The input data for the CNN training set consists of six-axis data (linear accelerations: *a_x_*, *a_y_*, *a_z_* and angular velocities: *ω_x_*, *ω_y_*, *ω_z_*) from the left and right feet, with each foot trained separately. Each foot has six features. The output labels of the training set are the calibrated XYZ-distances. The input layer size is (6, 1392). The kernel size is 41. The input data are first processed through a 1D CNN, followed by four 1D CNN and pooling layers, with the filter size growing from 64 to 1024, doubling at each layer. Then, it goes through four 1D CNN and unpooling layers, with the filter size decreasing from 1024 to 64, halving at each layer. Finally, a 1D CNN layer processes the output into XYZ coordinates of 1392 points.

Based on the walking distance–time curve of the X-distance, each step of a single foot’s walk can be segmented. Then, gait parameters such as step length, stride length, foot angle, walking speed, and the time difference between the left and right foot landing in the gait cycle can be calculated:

1. Gait Cycle Time: The time between two consecutive landings of the same foot;
2. One Step Move Time: The time between alternating landings of the left and right feet;
3. Gait Cycle Length: The step length between two consecutive landings of the same foot;
4. One Step Move Length: The distance between alternating landings of the left and right feet;
5. Angle At Stop: The foot angle when a single foot stops during a step.

Based on the steps of the object, we can use the form of (standard deviation, mean), (standard deviation, mean) to present the above parameters. The first set of data represents the standard deviation and mean of the left foot, and the second set represents the standard deviation and mean of the right foot. Taking one healthy participant and one post-stroke patient as examples, Table 5 shows a healthy person’s gait parameters, where the standard deviations are very small, and the average values of the left and right feet are not significantly different. Table 6 shows the gait parameters of a patient, where the standard deviations of the One Step Move Time for the left foot (0.075) and the right foot (0.115), the standard deviations of the Gait Cycle Length for the left foot (0.138) and the right foot (0.079), and the means of the One Step Move Length for the left foot (0.153) and the right foot (0.477) can be seen, and the standard deviations and mean of the Angle At Stop for the left foot (3.354, −11.985) and the right foot (0.545, −2.184) show significant differences. These parameters may provide some insights for scoring the G.A.I.T. in the future.

In the current work, we have derived a formula to score the G.A.I.T. based on the observations and the results from the gait parameters and GC’s states. We found the pitch angles of the left terminal stance, right terminal stance, left loading stance, and right pre-swing, and the times associated with these states are related to the G.A.I.T. score. Score 0 of G.A.I.T. represents normal walking. Therefore, the smaller the *p*-value (similar to the Gait Cycle Time) of Equation (18) is the less the difference between the walking of the left and right feet is. The closer the two variables of *c* in Equation (8) and *e* in Equation (4) are the more consistent the left foot is walking. Similarly, the same logic applies to variables *k* in Equation (10) and *g* in Equation (6), *f* in Equation (5) and *d* in Equation (9), and *h* in Equation (7) and *m* in Equation (11). The larger the value of *a* in Equation (14) is the more unbalanced the two feet are. The same logic applies to the value of *b* in Equation (15). The *q* and ${min(Arange}_{L}\;,{\;Arange}_{R})$ in Equation (20) are used as the bases to normalize the calculated score. Based on statistical analysis, the calculated scores and the doctor’s scores are significantly correlated with Pearson correlation = 0.752 and Spearman’s rho = 0.933 at the 0.001 level (two-tailed).

## 5. Conclusions

We have successfully developed and tested an IMU-based gait data acquisition system that is easy to install in a clinic and hospital and effective for post-stroke patients. Our approach includes a straightforward yet precise calibration process to mitigate IMU drifting errors and a CNN model to obtain real-time coordinates of the subject’s feet. The calibration process, tailored to the unique characteristics of 10 m linear walking, has yielded satisfactory results. The CNN model accurately predicts the coordinates in real time using six-axis data from each foot. By processing XYZ distances and pitch angles, we can easily determine the GC’s eight states and extract key parameters to calculate G.A.I.T. scores using our proposed formula. Statistical analysis reveals a significant correlation between the calculated G.A.I.T. scores and the doctor’s observational scores, with Pearson correlation at 0.752 and Spearman’s rho at 0.933, both significant at the 0.001 level (two-tailed). These findings underscore the reliability and potential of our system in clinical settings.

## 6. Patents

Based on this work, a patent, application # 113214518, has been filed in the Intellectual Property Office, Ministry of Economic Affairs, Taiwan, R.O.C.

## Figures and Tables

**Table 1:** G.A.I.T scores.

Subject #	Score by Doctor	Score by IMU
1	0	0.2732
2	4	0.7906
3	5	0.7266
4	3	0.3859
5	3	0.3620
6	5	0.5506
7	10	1.1644
8	6	1.1418
9	3	0.5846
10	3	0.4490
11	9	4.0168
12	9	2.3792
13	3	0.7701
14	0	0.3567
15	0	0.1239
16	0	0.2664
17	4	0.4509
18	0	0.1600
19	3	0.3820
20	0	0.2640
21	5	0.7700
22	0	0.2070
23	7	0.8810
24	3	0.4000
25	0	0.1640
26	0	0.1860

**Table 2:** Means and standard deviations of two sets of scores.

	Mean	Std. Deviation
Score by doctor	3.35	3.032
Score by IMU	0.7174	0.83552

**Table 3:** Pearson Correlation.

		Score by Doctor	Score by IMU
Score by doctor	Pearson Correlation	1.000	0.752
	Sig. (2-tailed)		0.000
Score by IMU	Pearson Correlation	0.752	1.000
	Sig. (2-tailed)	0.000	

**Table 4:** Spearman’s rho.

			Score by Doctor	Score by IMU
Spearman’s rho	Score by doctor	Correlation Coefficient	1.000	0.933
		Sig. (2-tailed)		0.000
	Score by IMU	Correlation Coefficient	0.933	1.000
		Sig. (2-tailed)	0.000	

**Table 5:** Gait parameters of a healthy person.

	Left Foot (std. dev., Mean)	Right Foot (std. dev., Mean)
Gait Cycle Time	(0.08744, 1.35300)	(0.03945, 1.34750)
One Step Move Time	(0.04715, 0.61750)	(0.01795, 0.71833)
Gait Cycle Length	(0.05711, 1.20995)	(0.02178, 1.33088)
One Step Move Length	(0.10636, 0.58199)	(0.10360, 0.66631)
Angle At Stop	(0.53145, 2.75407)	(0.94228, −1.17001)

**Table 6:** Gait parameters of a post-stroke patient.

	Left Foot (std. dev., Mean)	Right Foot (std. dev., Mean)
Gait Cycle Time	(0.12664, 1.85375)	(0.11065, 1.83958)
One Step Move Time	(0.07505, 0.86346)	(0.11515, 0.97821)
Gait Cycle Length	(0.13804, 0.69266)	(0.07912, 0.63808)
One Step Move Length	(0.18417, 0.15301)	(0.19998, 0.47711)
Angle At Stop	(3.35361, −11.98528)	(0.54522, −2.18445)

## Data Availability

The datasets presented in this article are not readily available because the data are part of an ongoing study.
